# Comparison of amino acid profiles and metabolic gene expression in muskrat scented glands in secretion and non-secretion season

**DOI:** 10.1038/srep41158

**Published:** 2017-02-01

**Authors:** Yimeng Li, Tianxiang Zhang, Mengyuan Fan, Juntong Zhou, Shuang Yang, Meishan Zhang, Lei Qi, Shaobi Lin, Defu Hu, Shuqiang Liu

**Affiliations:** 1 College of Nature Conservation, Beijing Forestry University, Beijing 100083, People’s Republic of China; 2 Zhangzhou Pien Tze Huang Pharmaceutical Co., Ltd., No.1 Shangjie Street, Zhangzhou City, Fujian Province 363700, People’s Republic of China.

## Abstract

The scented gland is an organ responsible for producing musk in muskrats. During musk secretion season, the metabolism of glandular cells increases in the scented glands and a large amount of musk is synthesised. In this study, we collected scented gland arterial blood from six healthy adult male muskrats during non-secretion season (November). We also obtained scented gland arterial blood, venous blood, and musk from six healthy adult males during secretion season (March). Qualitative and quantitative analyses of free amino acids in blood and musk were performed with an automated amino acid analyzer. Additionally, we employed RNA sequencing technology to study the expression patterns of amino acid metabolic pathways in scented glands. Amino acid profile analysis indicates that scented glands can concentrate amino acids during secretion season, and transcriptome analysis suggests that some amino acid metabolism-related genes undergo significant seasonal changes. In summary, scented gland amino acid metabolism displays seasonal differences. Elevated amino acid metabolic activity during secretion season sustains the glands’ secretory function.

The muskrat (*Ondatra zibethicus* L.) is a herbivorous fur animal adapted to aquatic life. It is the only species in the genus *Ondatra* (family Cricetidae). In male muskrats, a special organ, the scented gland, develops between the lower abdominal muscles and the skin, mostly in the area above the epididymis and on both sides of the scrotum. During breeding season, from March to October, the scented glands of adult male muskrats can produce a substance referred by Van Dorp *et al*.^1^ as American musk. Chemical analysis has indicated that this musk contains a variety of ingredients, such as muscone, normuscone, macrocyclic ketones, peptides, and amino acids^1^,^2^. Musk has various pharmacological activities, such as reducing heart rate^3^, decreasing myocardial oxygen consumption^3^, anti-inflammatory and analgesic properties^4^,^5^, antibacterial properties^6^, reducing arterial blood pressure^4^, and promoting growth^7^. At present, musk collected from farmed muskrats is of high economic value for the consumer, chemical, and pharmaceutical industries^4^.

Muskrat scented glands undergo significant cyclical changes. During musk secretion season (March–October), their volume increases significantly, producing large amounts of musk. During non-secretion season, by contrast, musk yield gradually declines, scented glands are replaced by adipose tissue, and musk secretion stops. During secretion season, the cellular metabolism of scented glands is enhanced, consuming large amounts of nutrients. An adequate supply of amino acids is paramount for maintaining the metabolism of glandular cells. Proteins taken up with the diet are broken up and absorbed through the digestive system. Subsequently, they are transported throughout the body in the form of free amino acids in the blood. These are utilized by cells as basic materials for building peptides and proteins.

In spite of the glands’ seasonal changes, very few studies have investigated the glands’ amino acid profile and metabolism. Here, we assessed for the first time the amino acid metabolism of muskrat scented glands by analysing changes in the concentration of amino acids in the blood and musk, and coupling them to transcriptome analysis of scented gland tissues.

## Results

### Amino acid content of scented glands’ arterial blood during secretion and non-secretion season

**Figure 1:**
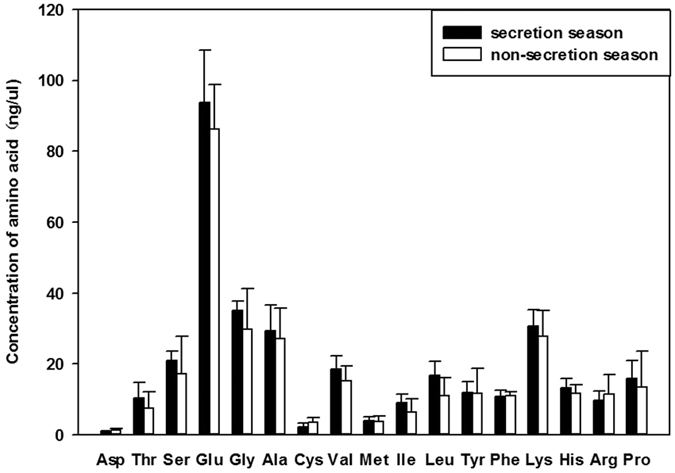
Concentration of amino acids in arterial blood during muskrat secretion and non-secretion season. Data are presented as means + SEM (n = 6). P > 0.05 (Student’s *t*-test).

A total of 17 common free amino acids were detected in the arterial blood from the scented glands during secretion and non-secretion seasons (values within parentheses, respectively) as listed below: six essential amino acids—isoleucine (9.16 ± 2.27 and 6.48 ± 3.76 ng/μL), leucine (16.81 ± 4.06 and 11.03 ± 5.07 ng/μL), lysine (30.61 ± 4.68 and 27.72 ± 7.29 ng/μL), phenylalanine (10.79 ± 1.73 and 10.94 ± 1.18 ng/μL), threonine (10.48 ± 4.33 and 7.55 ± 4.62 ng/μL), and valine (18.58 ± 3.79 and 15.30 ± 4.20 ng/μL); and 11 non-essential amino acids—alanine (29.44 ± 7.14 and 27.19 ± 8.48 ng/μL), aspartate (0.93 ± 0.20 and 1.29 ± 0.46 ng/μL), arginine (9.81 ± 2.52 and 11.53 ± 5.44 ng/μL), cysteine (12.19 ± 1.08 and 13.62 ± 1.32 ng/μL), glutamate (93.83 ± 14.64 and 82.24 ± 12.66 ng/μL), glycine (35.07 ± 2.57 and 29.90 ± 11.42 ng/μL), histidine (13.16 ± 2.71 and 11.68 ± 2.43 ng/μL), methionine (4.11 ± 1.07 and 3.76 ± 1.66 ng/μL), proline (15.99 ± 4.94 and 13.57 ± 9.99 ng/μL), serine (21.05 ± 2.65 and 17.17 ± 10.60 ng/μL), and tyrosine (12.01 ± 3.07 and 11.75 ± 6.93 ng/μL). Differences between arterial blood amino acid content during musk secretion and non-secretion season were not significant (P > 0.05) (Fig. 1).

### Analysis of amino acid content in musk and comparison to scented glands’ arterial blood content

**Figure 2:**
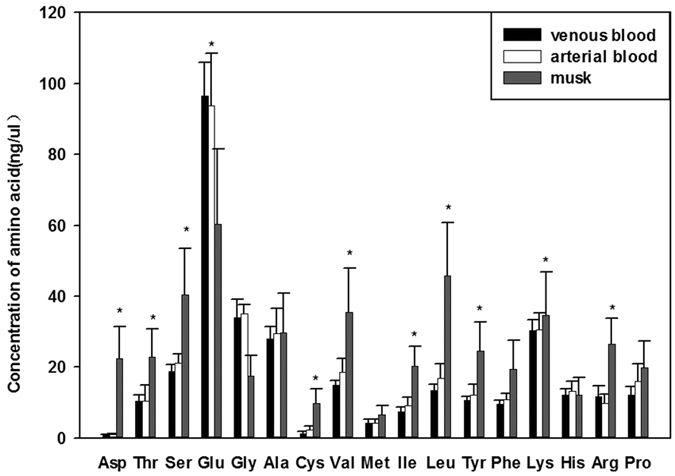
Concentration of amino acids in venous blood, arterial blood, and musk during secretion season. The concentration of amino acids in musk was significantly different from that in arterial blood. Data are presented as means + SEM (n = 6). *P < 0.05 (Student’s *t*-test).

A total of 17 common free amino acids were detected in musk during secretion season. These included six essential amino acids: isoleucine (20.18 ± 5.72 ng/μL), leucine (45.66 ± 15.00 ng/μL), lysine (34.59 ± 12.19 ng/μL), phenylalanine (19.35 ± 8.20 ng/μL), threonine (22.83 ± 7.88 ng/μL), and valine (35.46 ± 12.34 ng/μL); and 11 non-essential amino acids: alanine (29.62 ± 11.27 ng/μL), aspartate (22.33 ± 9.11 ng/μL), arginine (26.43 ± 7.29 ng/μL), cysteine (9.74 ± 4.16 ng/μL), glutamate (60.38 ± 21.08 ng/μL), glycine (17.41 ± 5.81 ng/μL), histidine (12.11 ± 5.00 ng/μL), methionine (6.57 ± 2.64 ng/μL), proline (19.89 ± 7.50 ng/μL), serine (40.34 ± 13.22 ng/μL), and tyrosine (24.62 ± 8.05 ng/μL). The level of ten of them (aspartate, arginine, cysteine, isoleucine, leucine, lysine, serine, threonine, tyrosine, and valine) was significantly higher in musk (Fig. 2) than in arterial blood (Fig. 1) during musk secretion season (P < 0.05).

### Amino acid profiles of scented glands’ venous and arterial blood during musk secretion season

A total of 17 common free amino acids were detected in scented glands’ venous blood during musk secretion season. These included six essential amino acids: isoleucine (7.37 ± 1.30 ng/μL), leucine (13.47 ± 1.68 ng/μL), lysine (30.34 ± 2.97 ng/μL), phenylalanine (9.51 ± 1.06 ng/μL), threonine (10.46 ± 1.73 ng/μL), and valine (14.81 ± 1.38 ng/μL); and 11 non-essential amino acids: alanine (27.96 ± 3.31 ng/μL), aspartate (0.83 ± 0.18 ng/μL), arginine (11.56 ± 3.20 ng/μL), cysteine (1.23 ± 0.50 ng/μL), glutamate (96.51 ± 9.42 ng/μL), glycine (34.03 ± 4.99 ng/μL), histidine (12.02 ± 1.84 ng/μL), methionine (4.19 ± 1.03 ng/μL), proline (12.00 ± 2.54 ng/μL), serine (18.72 ± 1.96 ng/μL), and tyrosine (10.69 ± 0.95 ng/μL). Amino acid content did not differ significantly between scented glands’ arterial (Fig. 1) and venous blood (P > 0.05) (Fig. 2) during secretion season.

### Transcriptome analysis of amino acid metabolism-related genes in muskrat scented glands’ tissues

**Figure 3:**
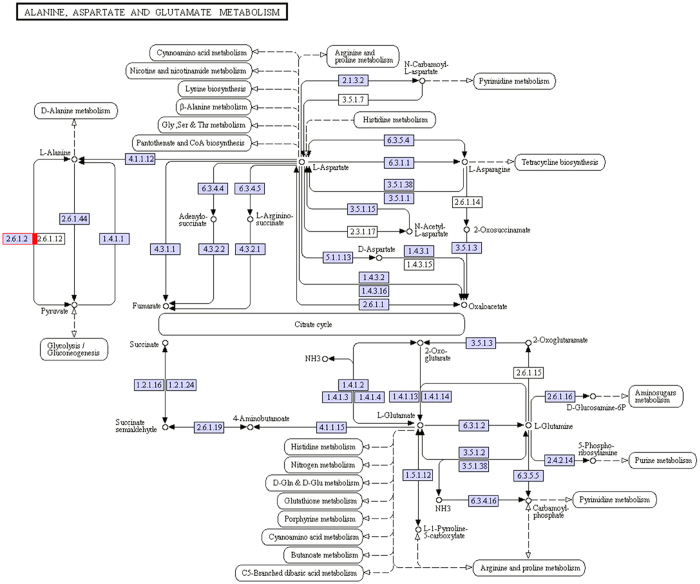
Schematic diagram showing alanine, aspartate, and glutamate metabolism.

**Figure 4:**
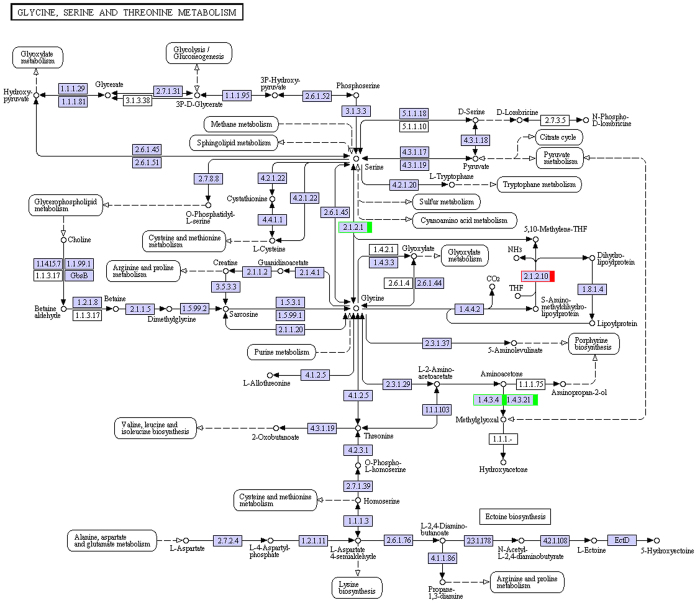
Schematic diagram showing glycine, serine, and threonine metabolism.

**Figure 5:**
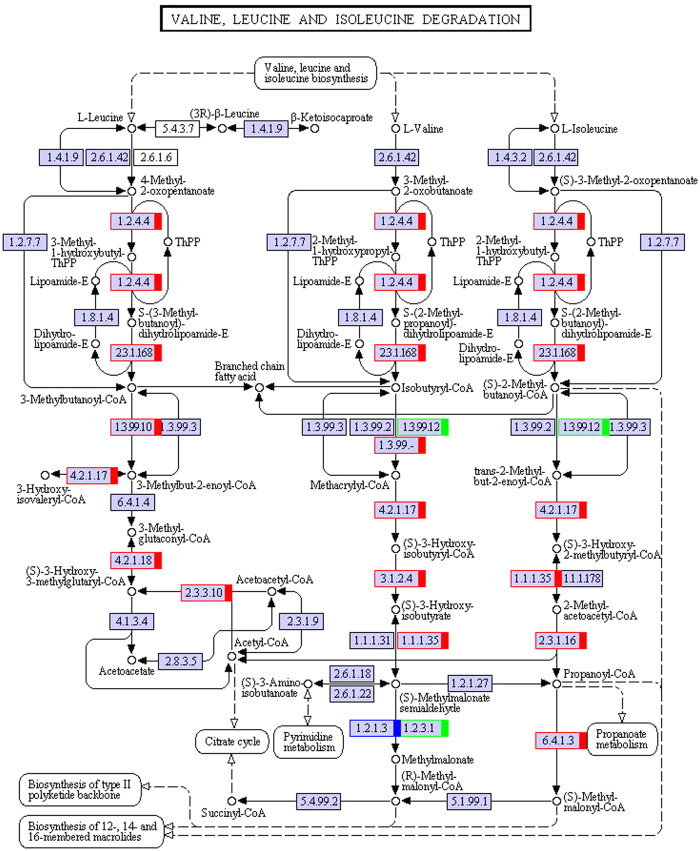
Schematic diagram showing valine, leucine, and isoleucine degradation.

**Figure 6:**
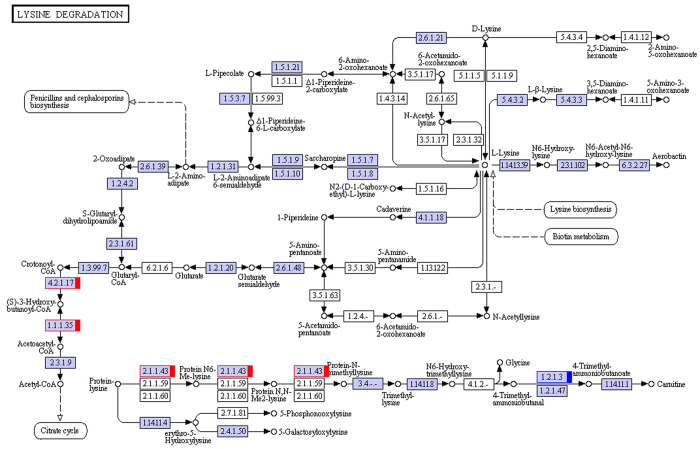
Schematic diagram showing lysine degradation.

**Figure 7:**
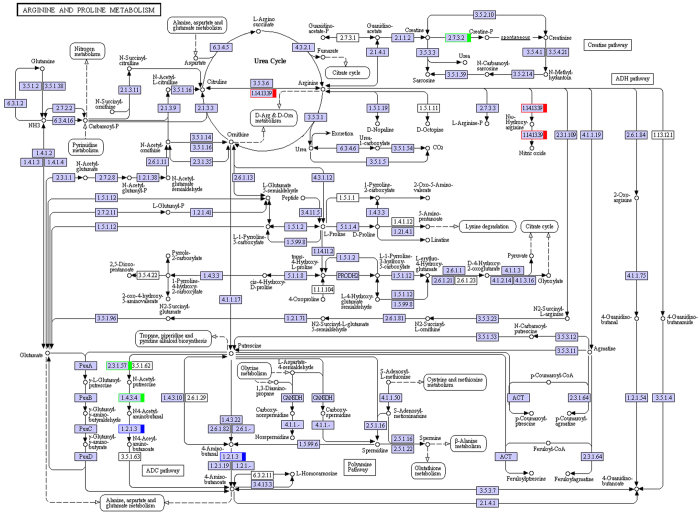
Schematic diagram showing arginine and proline metabolism.

We performed transcriptome analysis of muskrat scented glands’ tissues collected during secretion and non-secretion season. By comparing gene expression profiles of samples in different seasons, we identified differentially expressed genes, mapped them to the Kyoto Encyclopedia of Genes and Genomes (KEGG) database (http://www.genome.ad.jp/kegg/) reference pathways, and narrowed our attention to several crucial amino acid metabolic and signalling pathways. A total of 38 uni-transcripts were assigned to five pathways: alanine, aspartate, and glutamate metabolism (ko00250) (Fig. 3); glycine, serine, and threonine metabolism (ko00260) (Fig. 4); valine, leucine, and isoleucine degradation (ko00280) (Fig. 5); lysine degradation (ko00310) (Fig. 6); arginine and proline metabolism (ko00330) (Fig. 7). The red frame in the figures indicates that gene expression was up-regulated during secretion season, the green frame indicates that gene expression was down-regulated during secretion season, and the blue frame denotes genes whose expression was both up- and down-regulated. The analysis identified 24 up- and 14 down-regulated genes. Except for glycine, serine, and threonine metabolism (ko00260) signalling pathways, genes from other pathways were mainly up-regulated. The most relevant changes to the expression of genes involved in amino acid metabolic and signalling pathways are listed in Table 1.

**Figure 8:**
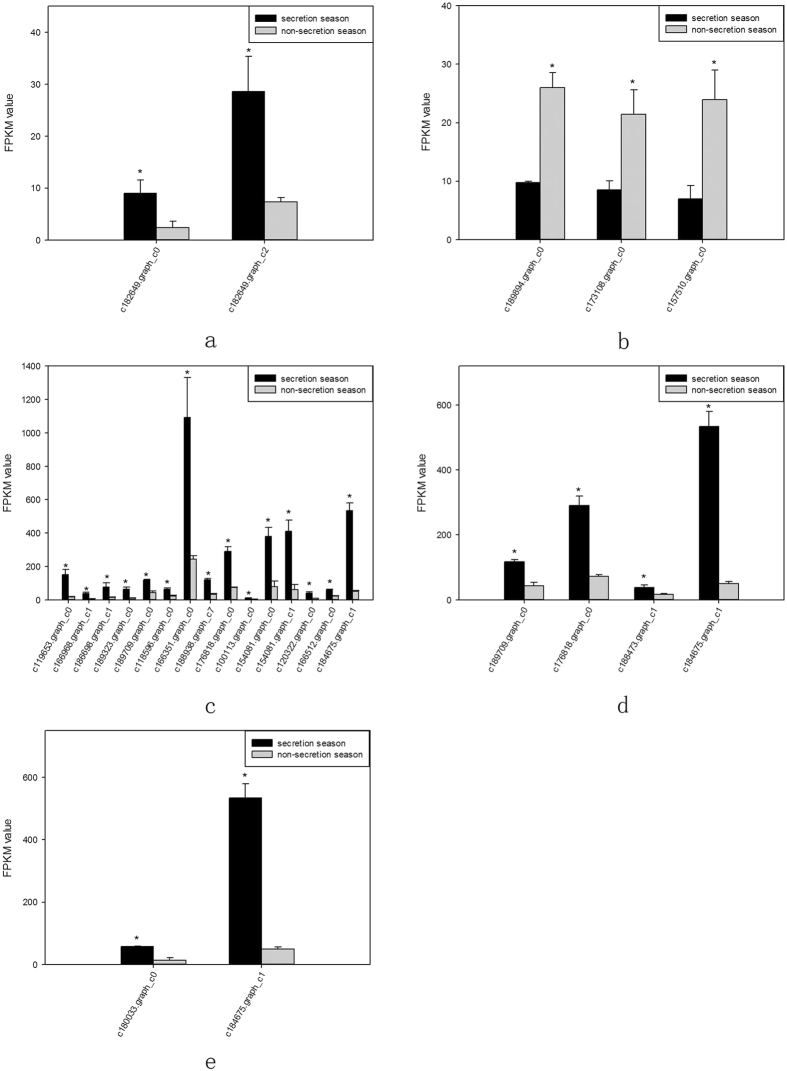
Accumulation of amino acid metabolism-related transcripts involved in musk production during secretion and non-secretion season. (**a**) Alanine, aspartate, and glutamate metabolism. (**b**) Glycine, serine, and threonine metabolism. (**c**) Valine, leucine, and isoleucine metabolism. (**d**) Lysine degradation. (**e**) Arginine and proline metabolism. Gene IDs are indicated. Data are presented as means + SD (n = 6). *FDR < 0.01 and Fold change ≥ 2. FDR, False Discovery Rate. FPKM, fragments per kilobase of transcript per million mapped reads.

The accumulation of amino acid metabolism-related transcripts involved in musk production during secretion and non-secretion season is detailed in Fig. 8.

### Validation of amino acid metabolism-related gene expression during secretion and non-secretion season

**Figure 9:**
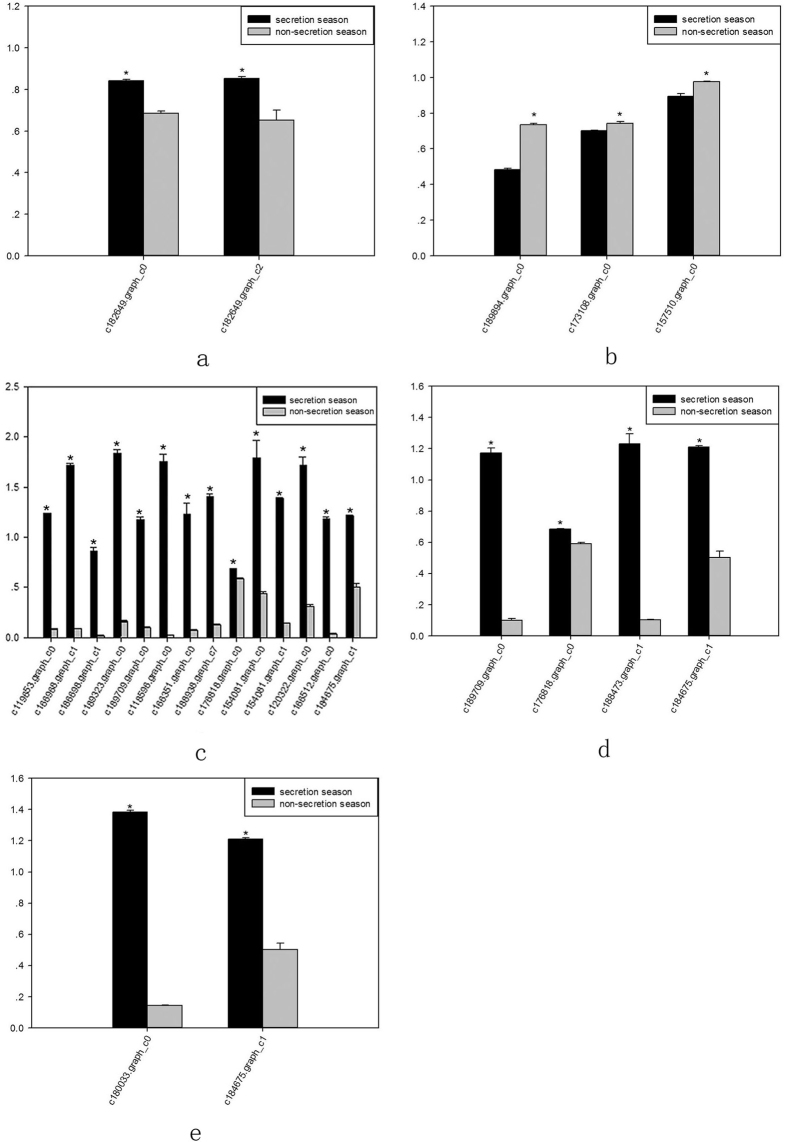
RT-PCR quantification of amino acid metabolism-related genes during secretion and non-secretion season. (**a**) Alanine, aspartate, and glutamate metabolism. (**b**) Glycine, serine, and threonine metabolism. (**c**) Valine, leucine, and isoleucine metabolism. (**d**) Lysine degradation. (**e**) Arginine and proline metabolism. Gene IDs are indicated. Data are presented as means + SD (n = 6). *P < 0.05 (Student’s *t*-test).

Based on transcriptome analysis results, we used reverse transcription PCR (RT-PCR) to validate the expression of amino acid metabolism-related genes. RT-PCR revealed alterations in the expression of genes from alanine, aspartate, and glutamate metabolism (ko00250); glycine, serine, and threonine metabolism (ko00260); valine, leucine, and isoleucine degradation (ko00280); lysine degradation (ko00310); and arginine and proline metabolism (ko00330) signalling pathways in the scented glands of muskrats during secretion and non-secretion season. These changes were consistent with transcriptome analysis results, indicating that our results reflected the actual expression of amino acid metabolism-related genes in scented glands. The results of RT-PCR corresponding quantification histograms are shown in Fig. 9 (P < 0.05).

## Discussion

Muskrat is a scent-secreting animal. The scented glands responsible for musk secretion undergo prominent cyclical changes. Here, we analysed for the first time the amino acid profiles of arterial blood, venous blood, and musk in scented glands and then used transcriptome analysis to study the muskrat scented glands’ metabolism.

We found that amino acid concentration in the arterial blood of scented glands did not change significantly between musk secretion and non-secretion season. If the concentration of amino acids in the blood increased dramatically with metabolic enhancement, this might affect other muskrat organs. Hence, maintaining a stable concentration of amino acids in the blood throughout secretion and non-secretion season ensures the overall physiological balance.

Next, we assessed amino acid concentration in musk and venous blood during secretion season, but found no significant differences in amino acid content between venous blood and arterial blood. Overall, amino acids were significantly more abundant in musk than in arterial blood, indicating that scented glands could concentrate amino acids in this organ. This finding is consistent with the results of a study on amino acids in bovine mammary glands and blood conducted by Wang *et al*.^8^. We speculate that this kind of enrichment in muskrat scented glands is slight and slow, so that physiological stability is ensured. In short, our results are in agreement with earlier studies describing alterations in amino acid metabolism in organs subjected to cyclical changes. Local amino acid enrichment can sustain demand from individual organs during special periods, without affecting the overall physiological balance.

To better understand the seasonal changes in amino acid metabolism, we performed transcriptome analysis and then selected metabolism-related differentially expressed genes. We found that most of the amino acids that were enriched in scented glands, such as alanine, leucine, valine, isoleucine, lysine, or arginine, had important physiological roles. Amino acid function is closely related to production of metabolic intermediates and energy generation. Two genes (c182649.graph_c0 and c182649.graph_c2) from the alanine, aspartate, and glutamate metabolism (ko00250) pathway were up-regulated during musk secretion season. They play an important role in converting glutamate to pyruvate. Alanine and pyruvate are the main substrates for the production of tryptophan and phenylalanine. Pyruvate plays also a central role in the inter-conversion of sugar, fat, and amino acids through acetyl-CoA and the citric acid cycle. Consequently, it is possible that an increase in pyruvate during musk secretion season provides more energy for glandular cells. Three genes (c189894.graph_c0, c173108.graph_c0, and c157510.graph_c0) from the glycine, serine, and threonine metabolism (ko00260) pathway were down-regulated during musk secretion season. This was likely because of methylglyoxal, a highly reactive dicarbonyl glycine metabolite that is often generated in the glycolytic pathway. Methylglyoxal can cause decreased mitochondrial function, depletion of antioxidant enzymes, and cross-linking with proteins in the form of advanced glycation end products, thereby leading to cytotoxicity^9^,^10^. Fifteen genes (c119653.graph_c0, c166968.graph_c1, c186698.graph_c1, c189323.graph_c0, c189709.graph_c0, c118596.graph_c0, c166351.graph_c0, c188938.graph_c7, c176818.graph_c0, c100113.graph_c0, c154081.graph_c0, c154081.graph_c1, c120322.graph_c0, c166512.graph_c0, and c184675.graph_c1) from the valine, leucine, and isoleucine degradation (ko00280) pathway were up-regulated during musk secretion season. They play important roles in catabolising these amino acids for energy production. Valine, leucine, and isoleucine are branched-chain amino acids (BCAA). BCAA are oxidized mostly in extrahepatic tissues^11^ and can generate ATP more efficiently than that by other amino acids. Thus, when the body uses proteins as a source of energy, BCAA are readily available^12^, particularly under specific physiological conditions. Four genes (c189709.graph_c0, c176818.graph_c0, c188473.graph_c1, and c184675.graph_c1) from the lysine degradation (ko00310) pathway were up-regulated during musk secretion season. They also play an important role in catabolising lysine for energy production. Lysine provides nitrogen for the synthesis of non-essential amino acids. Additionally, acetyl-CoA, a product of lysine degradation, contributes to energy generation through the citric acid cycle, the carnitine shuttle system, and fat metabolism, besides participating also in the immune response^13^,^14^ and BCAA metabolism. Two genes (c180033.graph_c0 and c184675.graph_c1) from the arginine and proline metabolism (ko00330) pathway were up-regulated during musk secretion season. They play an important role in increasing production of nitric oxide, a product of arginine metabolism that functions as a vasodilator and is beneficial for maintaining vascular permeability and ensuring a normal supply of amino acids from the blood^15^,^16^. Semi-quantitative RT-PCR demonstrated that changes in expression of genes related to alanine, glycine, BCAA, lysine, and arginine metabolism were consistent with results from transcriptome analysis. We speculate that changes in these genes are closely related to seasonal environmental changes. Overall, we observed significant changes in the metabolic pathways of six amino acids (alanine, arginine, isoleucine, leucine, lysine, and valine) in muskrat scented glands, which closely correlated with those amino acid profile results. The amino acid enrichment function performed by scented glands ensures that all local metabolic and energetic needs arising during secretion season are met. Alterations to the remaining amino acid metabolic pathways will require further research.

In summary, analyses of amino acid metabolic signalling pathways in muskrat scented glands will help clarify the glands’ regulatory mechanisms during musk secretion season. In addition, they may be used to improve the formulation of muskrat amino acid diets.

## Materials and Methods

### Animals and sample collection

Experimental muskrats were purchased from Jinmu Technologies (Xinji City, Hebei, China). In November (musk non-secretion season), six healthy adult male muskrats of similar size and weight were selected. Arterial blood samples were collected from the scented gland artery into collection tubes with anticoagulant and stored at −20 °C. In March (musk secretion season), six healthy adult male muskrats of similar size and weight were selected. Musk was collected from the scented glands and then stored in six bottles at 4 °C. Both venous and arterial blood was collected from the scented glands, divided into 12 tubes with coagulant, and stored at −20 °C. Scented gland tissue samples from both seasons were stored in liquid nitrogen for subsequent experimental analysis. The weight of the selected individuals was about 1.5 kg. All muskrats were killed by dislocation after anaesthesia.

All animals were treated in accordance with the National Animal Welfare Legislation. All experimental procedures were carried out in accordance with the guidelines on animal care established by Beijing Forestry University; the latter also approved the study.

### Amino acid analysis

To prepare degreased musk, a 200-μL sample was mixed with 400 μL water in a 1.5-mL microcentrifuge tube. The mixture was heated to 75 °C and incubated for 1 h until the sample became chyle-like. Subsequently, 600 μL anhydrous ether was added and mixed thoroughly before transferring the mixture to a 5-mL tube. The original microcentrifuge tube was rinsed with 3.3 mL anhydrous ether, which was then added to the sample mixture. The latter was left standing overnight at 25 °C. After the layers separated, the remaining 400–500 μL of water from the ether layer was removed and the tube was dried in a rotary evaporator. Following evaporation, an equal volume of 5% sulfosalicylic acid was added to the sample, the mixture was centrifuged at 15000 × *g* for 10 min, and the supernatant was analysed.

Arterial and venous blood was centrifuged at 2000 × *g* for 20 min. The upper serum (500 μL) was collected and 5% sulfosalicylic acid (0.025 g solid) was added. Samples were left standing to mix for 15 min, centrifuged at 15000 × *g* for 10 min, and the supernatant was collected for analysis (see below).

The following sample volumes were added to a Hitachi Amino Acid Analyzer (Tokyo, Japan): 420, 400, 410, 400, and 410 μL (degreased balsam); 400, 380, 390, 400, and 410 μL (blood). The analyzer was equipped with a separation column with ion-exchange resin #2622 PH (4L1426 Hitachi; 4.6 mm ID × 60 mm; 3-μm particles), operated at a column temperature of 57 °C and 0.35 mL/min flow rate. We also used a ninhydrin reaction column (4.6 mm ID × 40 mm), operated at a reaction temperature of 135 °C and 0.3 mL/min flow rate. Samples were detected at 570 nm and 440 nm (pro) using a visible spectrophotometer, with 20 μL injection volume and 50 min sample analysis time.

### Transcriptome analysis

Total RNA from each muskrat scented gland sample was isolated using TRIzol reagent (Qiagen, Valencia, CA, USA). Quantity and integrity of total RNA was assessed using an Agilent Bioanalyzer 2100 system (Agilent Technologies, San Diego, CA, USA) and 1% agarose gel electrophoresis; RNA concentration was measured using the Qubit RNA Assay Kit on a Qubit 2.0 Flurometer (Life Technologies, Carlsbad, CA, USA). High-quality RNA samples from muskrat scented glands were sent to Biomarker Technologies Corporation (Beijing, China) for construction and sequencing of cDNA libraries. Poly (A)^+^ RNA was enriched and purified using oligo (dT) magnetic beads and then broken into short fragments. Using these cleaved mRNA fragments as templates, first-strand cDNA was synthesized with the help of random hexamer primers and M-MuLV Reverse Transcriptase (RNase H-). Second-strand cDNA synthesis was subsequently performed using DNA Polymerase I and RNase H. Remaining overhangs were converted into blunt ends via exonuclease/polymerase activities, and were followed by purification of the double stranded cDNA with the AMPure XP system (Beckman Coulter, Beverly, MA, USA). Then, 3 μL USER Enzyme (NEB, USA) was used with size-selected, adaptor-ligated cDNA at 37 °C for 15 min followed by 5 min at 95 °C. PCR was performed using Phusion High-Fidelity DNA polymerase, universal PCR primers, and Index (X) Primer. PCR products were purified (AMPure XP system) and library quality was assessed on the Agilent Bioanalyzer 2100 system. Finally, paired-end sequencing was conducted on an Illumina HiSeq 2500 platform (Illumina Inc., San Diego, CA, USA) to generate 100-bp paired-end reads.

Raw data processing and base calling were performed by the Illumina instrument software. High-quality clean reads were obtained by removing adaptor sequences, duplicated sequences, ambiguous reads (‘N’), and low-quality reads. Then, the Q30 and GC-content were used to assess sequencing quality. In the absence of a reference genome, sequenced reads were assembled de novo using Trinity software with min_kmer_cov set to 2 by default and all other parameters also set to default.

Unigenes were annotated using BLAST (Basic Local Alignment Search Tool) searches against the Non-Redundant (NR), Swiss-Prot, KEGG, Gene Ontology (GO), Clusters of Orthologous Groups (COG), and Eukaryotic Orthologous Groups (KOG) databases with an E-value of 10^−5^ and HMMER software with an E-value of 10^−10^. Sample reads were compared with the transcript using Bowtie software. The information derived from the comparison was used by RSEM software to evaluate unigenes’ expression levels. The result was presented in terms of fragments per kilobase of transcript per million mapped reads (FPKM).

### RT-PCR

First-strand cDNA was from total RNA and was synthesised using genomic (g) DNA and FastQuant RT Enzyme from the RT FastQuant Kit (with gDNA) (Tiangen, Beijing, China). The 20-μL reaction mixture contained 260 ng of total RNA, 2 μL 5 × gDNA Buffer, 2 μL 10 × Fast RT Buffer, 1 μL RT Enzyme Mix, and 2 μL FQ-RT enzyme. The primer mix (25 μL) contained 6 μL of first-strand cDNA, 1 μL of each primer, 12.5 μL 2 × Taq PCR MasterMix, and 4.5 μL ddH_2_O (Tiangen, Beijing, China). Amplification conditions were as follows: initial denaturation of the RNA/cDNA hybrid at 94 °C for 3 min; then 33 cycles at 94 °C for 30 s, 55 °C for 20 s, and 72 °C for 20 s; and a final extension step at 72 °C for 5 min. An intron of the glyceraldehyde 3-phosphate dehydrogenase (*GAPDH*) cDNA fragment was amplified using primers: 5′- TTTGGCATCGTGGAAGGA-3′ (positions 508–525) and 5′-CGAAGGTAGAAGAGTGGGAGT-3′ (positions 892–872), and was used as internal control. The PCR products were separated on a 1% agarose gel and individual bands were visualized by ethidium bromide staining. All experiments were repeated three times.

### Statistical analysis

Results are presented as means + standard error of the mean (SEM) or standard deviation (SD). Student’s *t*-test was used for data analysis. A P value < 0.05 was considered statistically significant. In the selection of differentially expressed genes, we used a False Discovery Rate (FDR) < 0.01 and a Fold change ≥ 2 as standard. The FDR was obtained using the Benjamini–Hochberg method for correcting the P value.

## Additional Information

**How to cite this article**: Li, Y. *et al*. Comparison of amino acid profiles and metabolic gene expression in muskrat scented glands in secretion and non-secretion season. *Sci. Rep.* **7**, 41158; doi: 10.1038/srep41158 (2017).

**Publisher's note:** Springer Nature remains neutral with regard to jurisdictional claims in published maps and institutional affiliations.

## Figures and Tables

**Table 1:** Changes in expression of amino acid metabolism-related genes.

Unigene ID (EC name/Annotate)	Regulated	Log_2_FC
A. Alanine, aspartate, and glutamate metabolism (ko00250)-related genes.		
c182649.graph_c0 (2.6.1.2/GPT2)	Up	2.03
c182649.graph_c2 (2.6.1.2/alanine aminotransferase 2 isoform X1)	Up	2.16
B. Glycine, serine, and threonine metabolism (ko00260)-related genes.		
c155934.graph_c0 (2.1.2.10/aminomethyltransferase, mitochondrial isoform X1)	Up	1.50
c189894.graph_c0 (2.1.2.1/serine hydroxymethyltransferase, mitochondrial isoform -X1)	Down	−1.25
c173108.graph_c0 (1.4.3.4/amine oxidase)	Down	−1.17
c157510.graph_c0 (1.4.3.21/membrane primary amine oxidase)	Down	−1.65
C. Valine, leucine, and isoleucine degradation (ko00280)-related genes.		
c119653.graph_c0 (1.2.4.4/2-oxoisovalerate dehydrogenase subunit beta)	Up	3.40
c166968.graph_c1 (2.3.1.168/lipoamideacyltransferase componentof branched-chain alpha-keto acid dehydrogenase complex)	Up	2.87
c186698.graph_c1 (1.3.99.10/isovaleryl-CoA dehydrogenase)	Up	2.68
c189323.graph_c0 (1.3.99.-/isobutyryl-CoA dehydrogenase)	Up	2.84
c189709.graph_c0 (4.2.1.17/trifunctional enzyme subunit alpha)	Up	1.58
c118596.graph_c0 (4.2.1.18/methylglutaconyl-CoA hydratase)	Up	1.69
c166351.graph_c0 (2.3.3.10/hydroxymethylglutaryl-CoA synthase)	Up	2.35
c188938.graph_c7 (3.1.2.4/3-hydroxyisobutyryl-CoA hydrolase)	Up	2.04
c176818.graph_c0 (1.1.1.35/hydroxyacyl-coenzyme A dehydrogenase)	Up	2.15
c100113.graph_c0 (2.3.1.16/phosphatidylinositol 4,5-bisphosphate 5-phosphatase A isoform -X1)	Up	2.26
c154081.graph_c0 (2.3.1.16/3-ketoacyl-CoA thiolase)	Up	2.46
c154081.graph_c1 (2.3.1.16/3-ketoacyl-CoA thiolase)	Up	2.95
c120322.graph_c0 (6.4.1.3/ropionyl-CoA carboxylase alpha chain)	Up	2.37
c166512.graph_c0 (6.4.1.3/propionyl-CoA carboxylase beta chain)	Up	1.56
c184675.graph_c1 (1.2.1.3/fatty aldehyde dehydrogenase)	Up	3.62
c166519.graph_c0 (1.2.1.3/aldehyde dehydrogenase)	Down	−1.51
c188162.graph_c0 (1.2.1.3/retinal dehydrogenase 1-like)	Down	−1.63
c186399.graph_c0 (1.2.3.1/aldehyde oxidase-like)	Down	−1.86
c177257.graph_c0 (1.3.99.12/short/branched chain specific acyl-CoA dehydrogenase)	Down	−1.89
D. Lysine degradation (ko00310)-related genes.		
c189709.graph_c0 (4.2.1.17/trifunctional enzyme subunit alpha)	Up	1.58
c176818.graph_c0 (1.1.1.35/hydroxyacyl-coenzyme A dehydrogenase)	Up	2.15
c188473.graph_c1 (2.1.1.43/histone-lysine N-methyltransferase SETD7)	Up	1.34
c184675.graph_c1 (1.2.1.3/fatty aldehyde dehydrogenase)	Up	3.62
c166519.graph_c0 (1.2.1.3/aldehyde dehydrogenase)	Down	−1.51
c188162.graph_c0 (1.2.1.3/retinal dehydrogenase 1-like)	Down	−1.63
E. Arginine and proline metabolism (ko00330)-related genes.		
c180033.graph_c0 (1.14.13.39/nitric oxide synthase 3)	Up	2.31
c184675.graph_c1 (1.2.1.3/fatty aldehyde dehydrogenase)	Up	3.62
c172402.graph_c0 (2.3.1.57/diamineacetyltransferase 2)	Down	−1.75
c173108.graph_c0 (1.4.3.4/amine oxidase)	Down	−1.17
c153516.graph_c0 (2.7.3.2/creatine kinase B-type)	Down	−2.31
c166519.graph_c0 (1.2.1.3/aldehyde dehydrogenase, mitochondrial isoform -X1)	Down	−1.51
c188162.graph_c0 (1.2.1.3/retinal dehydrogenase 1-like)	Down	−1.63

FC: Fold change is a measure describing how much a quantity changes going from an initial to a final value; Log2FC: Expresses the FC of multiple values.
